# ﻿*Primulaweiliei* (Primulaceae), a new species from Hubei, Central China

**DOI:** 10.3897/phytokeys.242.119351

**Published:** 2024-05-09

**Authors:** Lin-Sen Yang, Zhi-Kun Wu, Hong-Wei Liang, Meng-Hua Zhang, Xian-Chun Zhang, Shuai Peng, Guang-Wan Hu

**Affiliations:** 1 Hubei Key Laboratory of Shennongjia Golden Monkey Conservation Biology, Administration of Shennongjia National Park, Shennongjia 442421, China Hubei Key Laboratory of Shennongjia Golden Monkey Conservation Biology, Administration of Shennongjia National Park Shennongjia China; 2 Guizhou University of Traditional Chinese Medicine, Guiyang, 550025, China Guizhou University of Traditional Chinese Medicine Guiyang China; 3 Key Laboratory of Plant genetics and Germplasm Innovation in the Three Gorges region, Three Gorges University / Biotechnology Research Center, Three Gorges University, Yichang, Hubei 443002, China Three Gorges University Yichang China; 4 Key Laboratory of Plant Resources Conservation and Utilization, College of Biology and Environmental Sciences, Jishou University, Jishou 416000, Hunan, China Jishou University Jishou China; 5 State Key Laboratory of Systematic and Evolutionary Botany, Institute of Botany, Chinese Academy of Sciences, Beijing 100093, China Institute of Botany, Chinese Academy of Sciences Beijing China; 6 CAS Key Laboratory of Plant Germplasm Enhancement and Specialty Agriculture, Wuhan Botanical Garden, Chinese Academy of Sciences, Wuhan 430074, China Wuhan Botanical Garden, Chinese Academy of Sciences Wuhan China; 7 Hubei Jiangxia Laboratory, Wuhan, 430200, China Hubei Jiangxia Laboratory Wuhan China

**Keywords:** China, conservation, *Primula*, Section *Aleuritia*, Shennongjia

## Abstract

In this study, we describe and illustrate a new species, *Primulaweiliei* L.S.Yang, Z.K.Wu & G.W.Hu, from the Shennongjia Forestry District, Hubei Province in Central China. It is morphologically assigned to Primulasect.Aleuritia based on its dwarf and hairless habit, long petiole, fruits longer than calyx and covered by farina on the scape. This new species is similar to *P.gemmifera* and P.munroisubsp.yargongensis in the same section, but it can be distinguished by its smaller calyxes, homostylous flowers, corolla tube throat without annular appendage and only 1–2 flowers in each inflorescence. Based on the assessment conducted according to the IUCN Red List criteria, we propose that *P.weiliei* be classified as a Critically Endangered (CR) species.

## ﻿Introduction

*Primula* L. (1753: 142) comprises approximately 500 species worldwide (POWO 2024), making it one of the largest genera in the family Primulaceae-Primuloideae. Most *Primula* species are distributed in the north temperate zones and alpine areas (Hu 1990, 1994). China, particularly the Eastern Himalaya-Hengduan mountains, is the diversity center of the genus, with approximately 300 species spanning 24 sections of *Primula* (Hu 1990, 1994). Hubei province in central China has recorded around 23 species, most of which are distributed in the western Hubei (Hu and Kelso 1996; Yang et al. 2020). The Shennongjia area has been extensively explored since the first visit by Augustine Henry in 1888 and Ernest Henry Wilson’s expedition from 1899 to 1911 (Wilson 1929). Recognized as one of the three centers of endemism for seed plants in China (Ying 1996), Shennongjia serves as the type locality for 659 vascular plant species (Xie and Xiong 2020), making it a significant area for biodiversity research and conservation.

In June 2019, during an investigation of a remote mountain peak in Shennongjia, we encountered an unknown plant belonging to the Primulasect.Aleuritia. We observed two distinct populations consisting of fewer than 50 individuals and collected flowering specimens. In August of the same year, we collected specimens at the fruiting stage, including seeds. In July 2020, we revisited the discovery site to collect additional specimens and capture further photographs of the flower.

According to the Flora of China, there are 49 species in Primulasect.Aleuritia, comprising 47 heterostylous, and 2 homostylous species viz. *Primulaclutterbuckii* Kingdon-Ward and *Primulameiotera* (W. W. Sm. & H. R. Fletcher) C. M. Hu (Hu 1990; Hu and Kelso 1996; Wu et al. 2013; Xu et al. 2017; Ju et al. 2023). The heterostylous species is characterized by pin flowers (with longer styles) and thrum flowers (with shorter styles), while the homostylous type lacks such differentiation, featuring only longer styles (Hu 1990; Hu and Kelso 1996).

Through thorough morphological examination of the newly collected materials, literature review, and comparison with potentially similar species, we have confirmed that this collection is undescribed and distinct from all currently known *Primula* species. Therefore, we describe and illustrate this collection as a new taxon to science named *Primulaweiliei* L.S.Yang, Z.K.Wu & G.W.Hu.

## ﻿Materials and methods

Initial morphological observations, measurements and descriptions of the new taxa were based on living individuals from the wild. Detailed photographs were also taken using a NIKON D750 camera during collection. Some living plants were harvested from the field and cultivated in the Shennongjia National Park lab to study further their growth process, morphological characteristics, and measurements. The type specimens were collected directly from the field, then dried, labelled and databased before being deposited in the Herbarium of Wuhan Botanical Garden (HIB) and Herbarium of the Institute of Botany, Chinese Academy of Sciences (PE). To identify the morphological differences with related species, we consulted relevant literature in the Flora of China and relevant specimens, including some type specimens from the PE, A, E and K, acronym according to Thiers (2016).

## ﻿Taxonomy

### ﻿ Primula weiliei

Taxon classificationPlantaeEricalesPrimulaceae

L.S.Yang, Z.K.Wu & G.W.Hu sp. nov.

05791AF2-236C-5AF5-BA43-92B7BB57449F

urn:lsid:ipni.org:names:77341545-1

Figs 1 , 2

#### Type.

China. Hubei Province: Shennongjia Forestry District, Shennongjia National Park, in rock crevices of *Abies* forest, alt. 2700–2900 m, 11 June 2019 (fl.), *Lin-Sen Yang 103* (holotype HIB!; isotype PE!).

#### Diagnosis.

This new species resembles *Primulagemmifera* and P.munroisubsp.yargongensis but differs significantly from the latter in several aspects: umbels are 1–2-flowered, scapes are 5–10 cm long, bracts measure 2–4 mm long, campanulate calyx is 3–4 mm long, flowers are homostylous, and the corolla throat lacks an annular appendage. Among the homostylous types in Primulasect.Aleuritia, *P.clutterbuckii*, *P.meiotera*, and *P.weiliei* share some similarities but are distinct from each other. *P.clutterbuckii* and *P.meiotera* feature multiple short stems with clustered leaves showing large and deep serrations along the leaf margin, along with a thick powder abaxially. In contrast, *P.weiliei* has a single rhizome, fewer leaves with fewer dentate or shallow teeth on the margin, and a slightly farinose abaxial surface.

**Figure 1.**
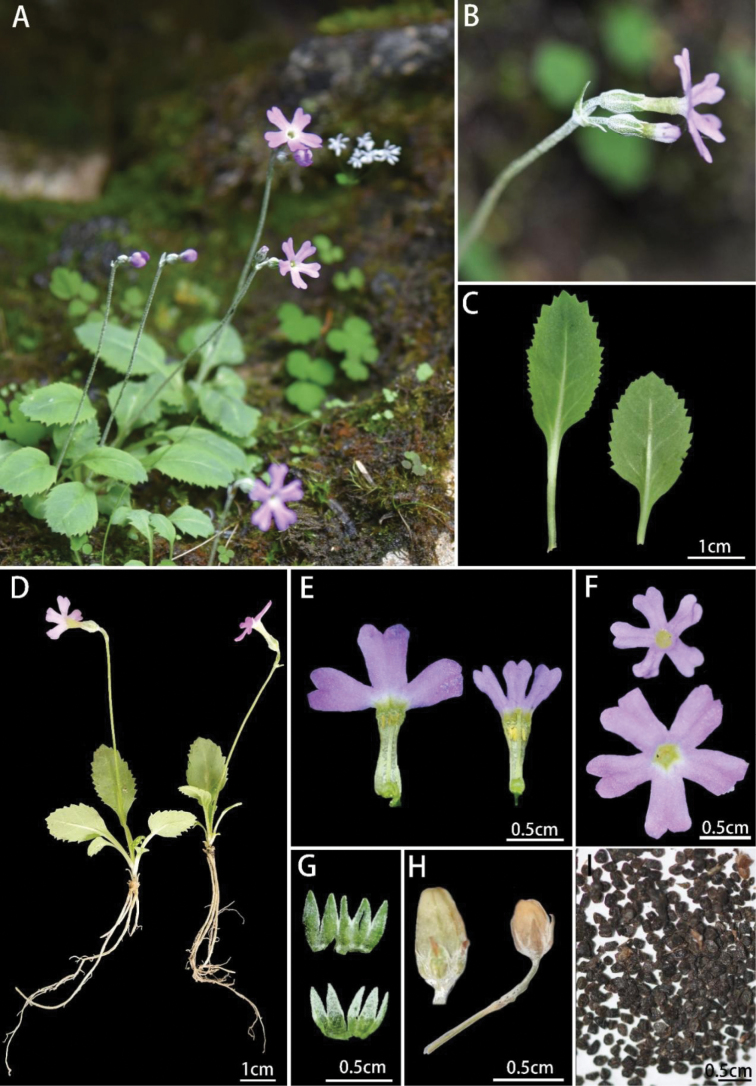
*Primulaweiliei* L.S.Yang, Z.K.Wu & G.W.Hu **A** habitat **B** inflorescence **C** leaves **D** habits **E** corollas, long homostyly **F** corollas adaxial surface **G** calyxes, the upper abaxial, the lower adaxial **H** fruits **I** seeds.

#### Description.

Perennial herbs, 3–5 cm tall, with a small rhizome and few fibrous roots. ***Leaves*** scale-like at the lower part, cauline on the upper part, 4–6; petiole 15–20 mm long, ca. 1/3–1× length of the blade, upper concave; blade spatulate, 13–23 × 8–18 mm, base attenuate, margin shallowly serrate to sharply incised-serrate, apex blunt, slightly farinose or none on both surfaces. Scape 5–10 cm long, obviously white farinose, 1–2(–3) flowered; bracts usually 2(when 1 flower)–3(when 2 flowers), linear-lanceolate, ca. 2–4 mm long, farinose on both surfaces, denser adaxially; pedicel 5–8 mm long, farinose. ***Flower*** homostylous; calyx campanulate, farinose on both surfaces, denser adaxially, tube *c.* 2 mm long, parted to middle, lobes 5, *c.* 2 mm long, lanceolate to triangular, apex mucronate or acuminate; corolla pink or purplish red, tube 7–9 mm long, tubular, narrowed in the middle, gradually widened apically, throat without annular appendage, mouth 2–3 mm wide, limb 10–14 mm wide, lobes obovate, 6–7 × 4 mm, slightly to deeply emarginate; stamen attached to near 3/4 of the tube, 1 × 0.2 mm, apex blunt, slightly lower of tube mouth; ovary nearly spherical, with a smooth surface, placentation free central, style 6 mm long, stigma spherical, *c.* 1 mm in diam., extending to tube apex. Capsule oblong, 4–7 × 3–4 mm, 1.5× to 2× as long as calyx; seeds 60–150, irregularly polygonal, light earthy yellow, black after exposure.

**Figure 2.**
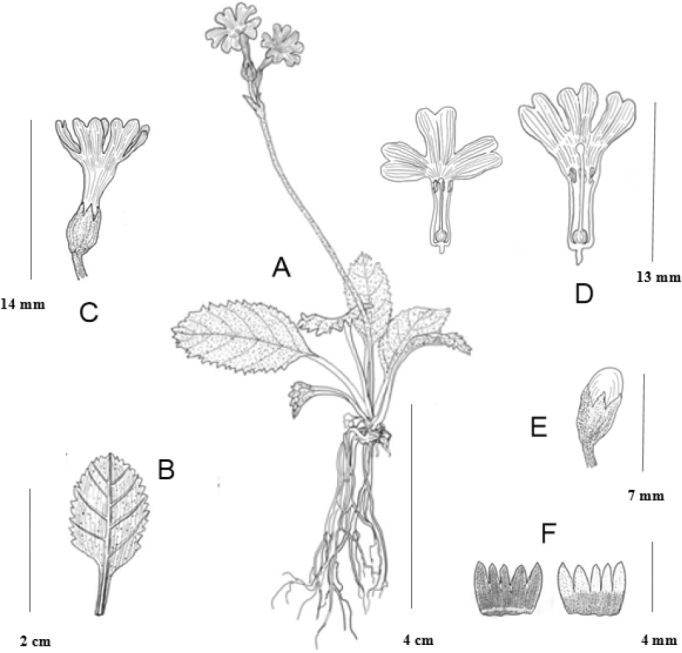
*Primulaweiliei***A** habit **B** leaf abaxial side **C** flower lateral view **D** corollas, long homostyly **E** ripe fruit **F** calyx, abaxial view (left), adaxial view (right). (Drawn by Ms Xiang-Li Wu).

#### Phenology.

Flowering occurs from June to July, and fruiting occurs from July to August.

#### Etymology.

The specific epithet ‘weiliei’ honours Prof. Chen Weilie, a plant ecologist and geobotanist in China, and the first director of the Shennongjia Biodiversity Positioning Research Station, Chinese Academy of Sciences.

#### Vernacular name.

Chinese Mandarin: wei lie bao chun (伟烈报春).

**Figure 3.**
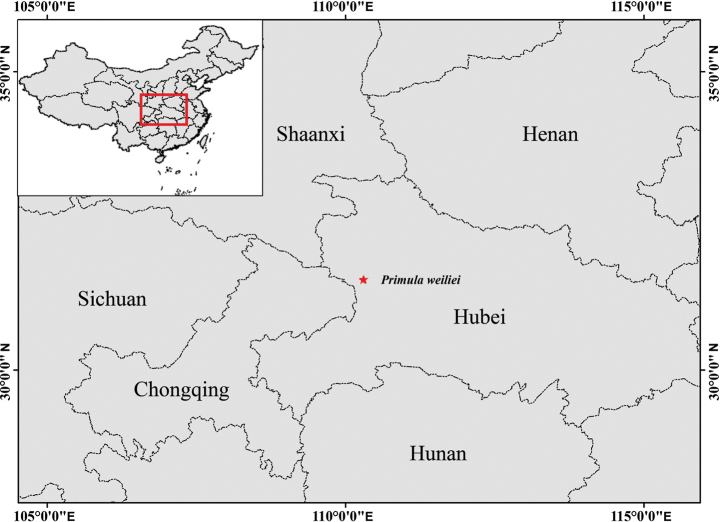
The distribution of *Primulaweiliei* (star).

**Distribution and habitat**. *Primulaweiliei* was found exclusively in Shennongjia National Park, located in Hubei, China (Fig. 3). It thrives in rock crevices near forests of *Abiesfargesii* Franch., while coexisting with other herbs and bushes, such as *Rhododendonconcinnum* Hemsl., *Juniperussquamata* Buch-Ham. ex D.Don, *Alliumcyaneum* Regel, *Loniceratangutica* Maxim and *Androsacehenryi* Oliv.

#### Conservation status.

*Primulaweiliei* is an exceedingly rare species found in only two small, closely situated populations, with a total population size of approximately 50 individuals. According to the IUCN Red List Categories and Criteria (IUCN 2022), it meets the criteria for being classified as a Critically Endangered species (CR A2acde; B2ab; C2a(i)). Given the threats of climate change and animal damage contributing to population decline, it is imperative to monitor these populations closely to prevent further degradation or extinction.

#### Additional specimens examined.

*Primulagemmifera*: China, Sichuan, 11500 ft, July 1904, *E. H. Wilson, 4031* (A barcode A00073544, image seen!); Yunnan, 14200 ft, Aug 1914, *George Forrest 13231* (K barcode K000750072, image seen!). *Primulameiotera*: China, Xizang, Kongbo, 11500 ft, 12 Jul 1938, *F. Ludlow, G. Sherriff, & G. Taylor 5218* (BM barcode BM000996905, E barcode E00024506, images seen!). *Primulaclutterbuckii*: India, Bangladesh & Pakistan, [ca. 28°21'N, 96°37'E], 10000–11000 ft, 23 May 1928, *F. Kingdon-Ward 8235* (E barcode E00024452, K barcode K000750416, images seen!).

#### Taxonomic notes.

We have thoroughly compared the new species and several closely related species, as detailed in Table 1. Notably, *Primulaweiliei* and its closely related species exhibit similar characteristics, including irregularly denticulate leaf margins, winged leaf stalks, lanceolate bracts, campanulate calyx typically splitting to the middle or below, lanceolate sepals, and generally purple corollas. However, a few distinct characteristics can differentiate the new species from its closely related species. For instance, the calyx length of *P.gemmifera* and P.munroisubsp.yargongensis exceeds that of the new species by double its length. Furthermore, while the latter species typically bear more than two flowers on one scape, the new species only bear 1–2 flowers. Additionally, both the latter species are heterostylous with throat appendages in the corolla tubes, making them easily distinguishable (Hu 1990; Hu and Kelso 1996). The scarcity of homostylous types in Primulasect.Aleuritia underscores the importance of their characteristics. Despite many similarities among the three known species with homostylous flowers, such as the presence of 1–2 flowers in the inflorescence, sepals splitting in half, and the stigma and anther tips located near the corolla tube mouth, notable differences exist among these species. For instance, *P.weiliei* has sparse leaf teeth, scarcely farinose leaves, and a 7–9 mm corolla tube length. In contrast, *P.clutterbuckii* and *P.meiotera* have corolla tubes that are 15–16 mm and 4 mm long, respectively, and their leaves have coarse marginal teeth and are densely farinose on the abaxial surface.

**Table 1.** Morphological comparison between *Primulaweiliei* and its related species.

Characters	*P.weiliei*	P.munroisubsp.yargongensis	*P.gemmifera*	*P.clutterbuckii*	*P.meiotera*
Plants farinose	Farinose	Efarinose	Farinose	Farinose	Farinose
Scape length	5–10 cm	10–30 cm	8–30 cm	0.8–2.5 cm	0.3–4 cm
Inflorescence	1–2(3)-flowered	2–6-flowered	3–10-flowered	1–2-flowered	1–2-flowered
Bracts bottom prolonged	Not	Bottom prolonged into auricles	Not	Not	Not
Calyx length	3–4 mm	5–10 mm	6–10 mm	8–10 mm	4 mm
Style	Homostylous	Heterostylous	Heterostylous	Homostylous	Homostylous
Corolla tube length	7–9 mm	10–12 mm	8–13 mm	15–16 mm	4 mm
Corolla throat appendage	Absent	Existing	Existing	Absent	Absent

Shennongjia, located in central China, is recognised as a biodiversity hotspot that has garnered the attention of numerous botanists, increasing botanical surveys conducted in the area in recent years. The discovery of *Primulaweiliei* further expands the known distribution of Primula species within this region, emphasising the significance of continual botanical exploration within biodiversity hotspots. Notably, the four species mentioned above, P.munroisubsp.yargongensis, *P.gemmifera*, *P.clutterbuckii*, and *P.meiotera*, closely related to *P.weiliei*, are distributed in southeastern Tibet and the Hengduan Mountains. This suggests a potential correlation between the plant floras of western Hubei and eastern Himalaya, necessitating further analysis involving a broader range of plant taxa to establish a confirmation. Given the limited number of known specimens originating from its type locality, it is imperative to expedite an investigation into the conservation status of this newly discovered species. Accordingly, the development and implementation of effective conservation strategies are pivotal to ensure the long-term survival of this species.

## Supplementary Material

XML Treatment for Primula weiliei

## ﻿Additional information

### Author ORCIDs

Lin-Sen Yang https://orcid.org/0000-0003-2410-6435

Zhi-Kun Wu https://orcid.org/0000-0002-4583-7782

Hong-Wei Liang https://orcid.org/0009-0002-0418-2561

Meng-Hua Zhang https://orcid.org/0000-0001-5326-6994

Xian-Chun Zhang https://orcid.org/0000-0003-3425-1011

Shuai Peng https://orcid.org/0000-0002-8064-920X

Guang-Wan Hu https://orcid.org/0000-0001-7728-7976

### Data availability

All of the data that support the findings of this study are available in the main textn.
